# Determination of dehydroepiandrosterone and its biologically active oxygenated metabolites in human plasma evinces a hormonal imbalance during HIV-TB coinfection

**DOI:** 10.1038/s41598-018-24771-8

**Published:** 2018-04-27

**Authors:** María Belén Vecchione, Javier Eiras, Guadalupe Verónica Suarez, Matías Tomás Angerami, Cecilia Marquez, Omar Sued, Graciela Ben, Héctor Miguel Pérez, Diego Gonzalez, Patricia Maidana, Viviana Mesch, María Florencia Quiroga, Andrea Claudia Bruttomesso

**Affiliations:** 1 Consejo Nacional de Investigaciones Científicas y Técnicas (CONICET) - Universidad de Buenos Aires, Instituto de Investigaciones Biomédicas en Retrovirus y Sida (INBIRS), Buenos Aires, Argentina; 2 Consejo Nacional de Investigaciones Científicas y Técnicas (CONICET) - Universidad de Buenos Aires, Unidad de Microanálisis y Métodos Físicos aplicados a la Química Orgánica (UMYMFOR), Facultad de Ciencias Exactas y Naturales, Buenos Aires, Argentina; 3 Centro de Alta Tecnología Analítica (CATA), Analytical Technologies S.A., Buenos Aires, Argentina; 4 Fundación Huésped, Buenos Aires, Argentina; 5 Hospital Juan A. Fernández, Buenos Aires, Argentina; 6 Universidad de Buenos Aires, Facultad de Farmacia y Bioquímica, Departamento de Bioquímica Clínica – INFIBIOC, Buenos Aires, Argentina

## Abstract

An estimated one third of the world’s population is affected by latent tuberculosis (TB), which once active represents a leading cause of death among infectious diseases. Human immunodeficiency virus (HIV) infection is a main predisposing factor to TB reactivation. Individuals HIV-TB co-infected develop a chronic state of inflammation associated with hypothalamic-pituitary-adrenal (HPA) axis dysregulation. This results in a hormonal imbalance, disturbing the physiological levels of cortisol and dehydroepiandrosterone (DHEA). DHEA and its oxygenated metabolites androstenediol (AED), androstenetriol (AET) and 7-oxo-DHEA are immunomodulatory compounds that may regulate physiopathology in HIV-TB co-infection. In order to study possible changes in plasma levels of these hormones, we developed an approach based on high performance liquid chromatography**-**tandem mass spectrometry (HPLC-MS/MS). To our knowledge, this represents the first report of their simultaneous measurement in HIV-TB individuals and the comparison with healthy donors, obtaining statistically higher plasma levels of DHEA, AET and 7-oxo-DHEA in patients. Moreover, we found that concentrations of 7-oxo-DHEA positively correlated with absolute CD4+ T cell counts, nadir CD4+ T cell values and with individuals who presented TB restricted to the lungs. This research contributes to understanding the role of these hormones in HIV-TB and emphasizes the importance of deepening their study in this context.

## Introduction

Tuberculosis (TB), a disease caused by the intracellular pathogen *Mycobacterium tuberculosis* (*Mtb*), is one of the ten leading causes of death worldwide, outweighing the number of demises attributed to human immunodeficiency virus (HIV)/AIDS. People infected with *Mtb* have a lifetime risk of progression to active TB of around 10%, while individuals with a compromised immune system, such as in the case of HIV infection, are 17–22 times more likely to develop the disease^1^. Around 14 million people are estimated to be co-infected with TB and HIV, together emerging as the leading infectious diseases in resource-limited countries^2,3^.

People co-infected with HIV-TB present chronically elevated pro-inflammatory cytokines which lead to hypothalamic-pituitary-adrenal (HPA) axis dysregulation^4,5^. Several cytokines can cross the blood-brain barrier and access the central nervous system, modulating hormone secretion^6,7^. These changes have been thoroughly studied in HIV, where HPA disorders may cause subclinical adrenal insufficiency leading to increased morbidity and mortality^8,9^. Furthermore, many of the anti-retroviral drugs can contribute to HPA axis dysfunction^8^. Regarding *Mtb*-infected individuals, it was reported that patients exhibit altered plasma levels of cortisol, prolactin, growth hormone, thyroid hormone, testosterone and dehydroepiandrosterone (DHEA)^10^. Together, the endocrine disturbances found in TB patients are related with worsened clinical status and unfavorable disease outcome^11^. Despite these data, reports about HPA imbalance during HIV-TB co-infection are very scarce. In HIV-TB individuals, an increased cortisol/DHEA ratio might lead to infection progression by inducing a shift from Th1 to Th2 immunologic responses^12^. Although the cross-talk between the immune and endocrine systems is responsible for an effective immune response against infectious agents^13^, the relevance of immune-endocrine interactions has not been fully elucidated yet.

DHEA is a hormone secreted mainly by the adrenal cortex, but also by the gastrointestinal tract, gonads, and brain. It serves several functions in the human body, which are classically associated with age-related changes such as metabolism alterations, cardiovascular disease, fertility and neuronal function^14–16^. However, it is now clear that DHEA is a regulator of immune functions, as it modulates the production of inflammatory cytokines, increases resistance to infections, exhibits antiviral activity and counteracts the immune-suppressive effects of glucocorticoids^17–21^. In previous studies, we demonstrated that DHEA modulates the immune response against *Mtb*, enhancing the cytotoxic Th1 and CD8+ T cell responses and negatively regulating the expression of transcription factor FoxP3. In line with this, we observed that DHEA enhanced *Mtb*-specific Th1 responses from human dendritic cells *in vitro*^12,22,23^.

The pleiotropic effects of DHEA might be explained through its direct interaction with specific receptors and/or its transformation into multiple metabolites^24^. On this issue, previous studies have reported that androstenediol (AED) and androstenetriol (AET) upregulate host immunity, conferring protection against bacterial and viral infections in a mouse model. Moreover, AET treatment increases CD4+/CD8+ T lymphocyte ratio after irradiation and the levels of IL-2, IL-3, and IFN-γ, counteracting hydrocortisone immune suppression^17,25–28^. Additionally, 7-oxo-dehydroepiandrosterone (7-oxo-DHEA) has been reported as a thermogenic and neuroprotective compound^29,30^, although its effects on the immune system have not been studied in depth. As this compound is not a precursor of biologically active androgens or estrogens, it is considered that it could substitute DHEA in replacement therapies^31–33^.

In order to study adrenal imbalance during HIV-TB co-infection, we developed a specific approach based on HPLC–MS/MS technology for simultaneous determination of DHEA, AED, AET and 7-oxo-DHEA levels in human plasma samples. This involved the use of an internal standard (IS) and a sensitive measurement without sample derivatization. Furthermore, we compared the results from our approach with a validated radioimmunoassay. Remarkably, the results obtained in this work revealed biological relevance, as we found statistically significant differences between groups when comparing the levels of DHEA, AET and 7-oxo-DHEA in a cohort of healthy donors (HD) with those found in HIV-TB co-infected patients. Moreover, we observed that 7-oxo-DHEA levels were related with clinical parameters associated with disease outcome.

## Results

Among the main findings of this research: (1) The HPLC-MS/MS-based approach described here proved a sensitive and specific methodology for measuring the steroids under study; (2) The use of an IS prevented interferences in extraction caused by differences in matrix complexities between HIV-TB and HD; (3) Notably, HIV-TB patients exhibited higher plasma levels of DHEA, AET and 7-oxo-DHEA compared with HD; 4) 7-oxo-DHEA levels were positively related with absolute CD4+ T cell counts, CD4+ T cell nadir values and the development of pulmonar instead of extrapulmonar TB.

### HPLC-MS/MS approach for detection of steroid hormones: performance and validation results

**Figure 1:**
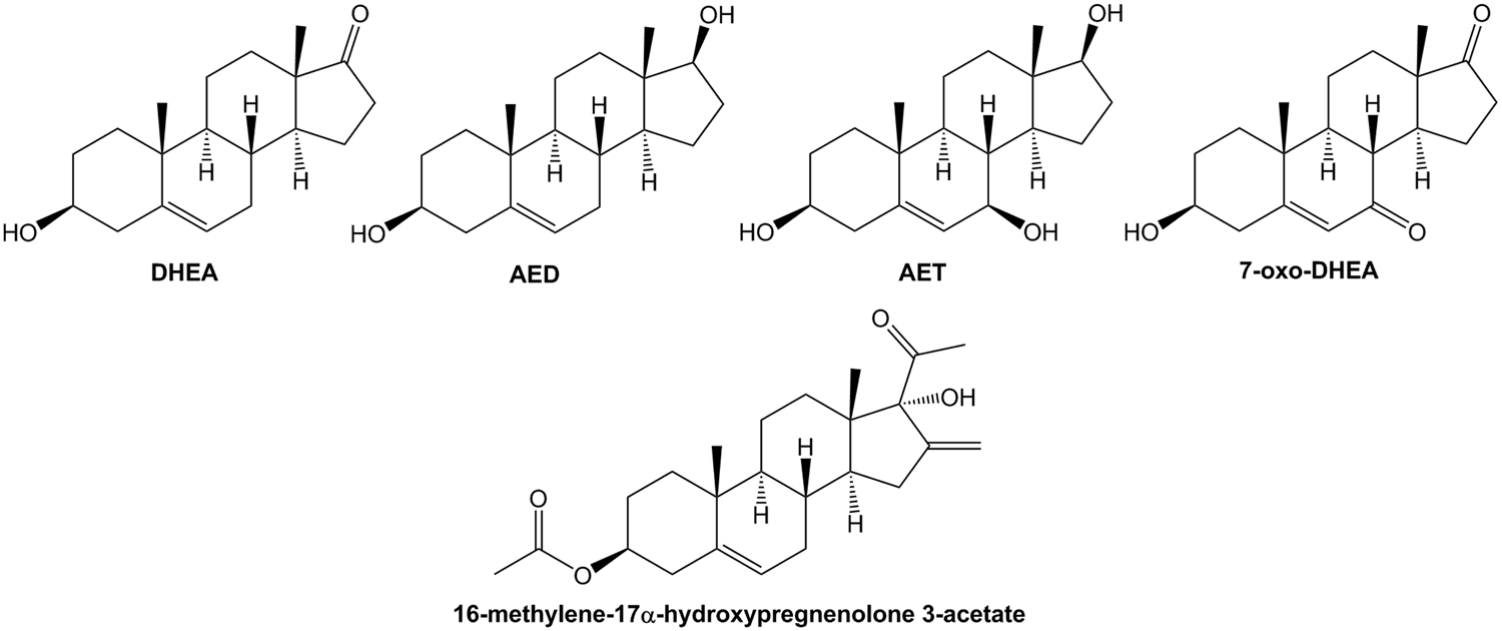
Chemical structure of the analytes studied. The hormones dehydroepiandrosterone (DHEA), androstenediol (AED), androstenetriol (AET), 7-oxo-DHEA and the compound used as an internal standard (IS) are represented.

We selected liquid chromatography-electrospray ionization tandem mass spectrometry as a tool to determine DHEA, AED, AET and 7-oxo-DHEA (Fig. 1) in human plasma samples. We used the method of multiple reaction monitoring (MRM) as a highly sensitive and selective technique for the quantitation of analytes in a complex biological matrix. In Table 1, we summarize the precursor and fragment ions, the optimized collision energy values and the retention times obtained in both the reference laboratory and the secondary laboratory. We evaluated the specificity of the method by comparing the chromatograms obtained from the analytes in a solution of MeOH with an extracted plasma sample, both fortified with standards and IS. Supplementary Fig. S1 depicts good chromatographic quality without significant interferences, giving a measurable response at the corresponding retention times of the ions monitored. Therefore, it can be concluded that the approach developed here was specific for the compounds studied and did not result in artifacts or interfering peaks.

**Table 1:** Multiple reaction monitoring transitions and retention times.

Analyte	Q1 Mass (Da)	Q3 Mass (Da)	Collision energy (V)	Retention Time A (min)	Retention Time B (min)
DHEA	271.1	253.1^#^	13	14.04	8.34
	271.1	197.1	20		
AED	273.1	255.1^#^	13	12.38	6.79
AET	289.2	271.4^#^	5	2.25	2.24
	289.2	253.2	14		
7-oxo-DHEA	303.2	285.2^#^	17	6.75	4.37
	303.2	81.3	29		
IS	369.1	309.3^#^	5	25.56	12.95
	369.1	251.3	13		

^#^Indicates ion quantified. Retention Time A corresponds to laboratory of reference (Thermo Fisher Scientific Inc. Ultimate 3000 RSLC Dionex HPLC) and Retention Time B to secondary laboratory (Agilent 1290 Infinity II LC with a ZORBAX Rapid Resolution High Definition).

Matrix effects can cause disturbances in the slope of a calibration curve^34^. Therefore, we evaluated possible interferences by comparing the results from both solvent-treated and fortified pooled plasma calibration curves, with results showing no statistically significant differences between both curve slopes. Also, we calculated matrix effect (ME) by assessing the MS/MS *response* of analytes into a MeOH solution and a pooled plasma extract, obtaining values near 100% when relative recoveries were evaluated. These data indicate that the responses in both solutions were similar and that possible matrix effects during the process were compensated by the use of the IS added before extraction (Table 2). Thus, we decided to use external calibration for the validation analysis and analyte assessment in plasma samples. Finally, in order to demonstrate a proportional relationship between the area ratios versus analyte concentration, we determined the linearity of the assay and observed high linear responses along the calibration range (3–3000 ng/ml, R^2^ > 0.994).

**Table 2:** Validation results for the novel HPLC-MS/MS-based approach.

Analyte	ME (%)	Linearity	LOD (ng/ml)	LOQ (ng/ml)	Accuracy (RE%)	Precision Intraday (CV%)	Precision Interday (CV%)	Relative R (%)	Absolute R (%)	PE (%)
DHEA	107	0.999	1	4	19.0 14.4 10.7	3.2 2.7 7.5	20.1 2.9 7.0	102	72	109
AED	96	0.997	3	8	−19.9 −5.1 1.9	11.9 2.6 11.0	16.1 5.8 7.6	105	70	101
AET	110	0.995	6	18	−12.2 −14.7 −4.4	17.3 6.0 6.1	15.9 8.0 4.8	99	75	109
7-oxo-DHEA	98	0.994	5	16	−19.7 12.9 14.9	7.6 6.8 10.1	20.1 5.9 9.9	109	73	106

Matrix effect (ME), linearity, limit of detection (LOD), limit of quantification (LOQ), accuracy, intra and interday precision, relative and absolute recoveries (R) and process efficiency (PE) for each steroid. Accuracy and precision were indicated for QC at LLOQ (top), LQC (middle) and HQC (bottom).

The limit of detection (LOD) is defined as the lowest analyte concentration that can be detected and distinguished from the noise level but not necessarily quantified, while the limit of quantitation (LOQ) represents the lowest concentration of analyte that can be determined quantitatively with an acceptable level of precision^34^. We achieved a LOD ranging between 1 and 6 ng/ml, while the LOQ varied from 4 to 18 ng/ml (Table 2). In a previous report using radioimmunoassay (RIA), we determined that DHEA plasma levels ranged from 0.5 to 10 ng/ml^12^. Taking these data and the LOQ obtained for DHEA into account, we concentrated the sample by taking the initial 1000 μl of plasma in 100 μl of MeOH prior injection, therefore reaching the adequate concentration for the assay and demonstrating the accuracy and precision of analyte determination in our biological matrix. These parameters were calculated at 3 ng/ml as the lower limit of quantitation (LLOQ), 10 ng/ml as the lower quality control (LQC) and 3000 ng/ml as the higher QC concentrations from data obtained during 5-day validation (Table 2). In spite of the fact that the LOD for AET and 7-oxo-DHEA were slightly above the lowest point of the standard curve (3 ng/ml), this very low signal also exhibited precision and accuracy values that were in concordance with FDA guidelines^35^ (Table 2). Although further validation is required, we consider this methodology appropriate for the quantification of DHEA, AED, AET and 7-oxo-DHEA from human plasma samples and potentially suitable for clinical purposes.

**Figure 2:**
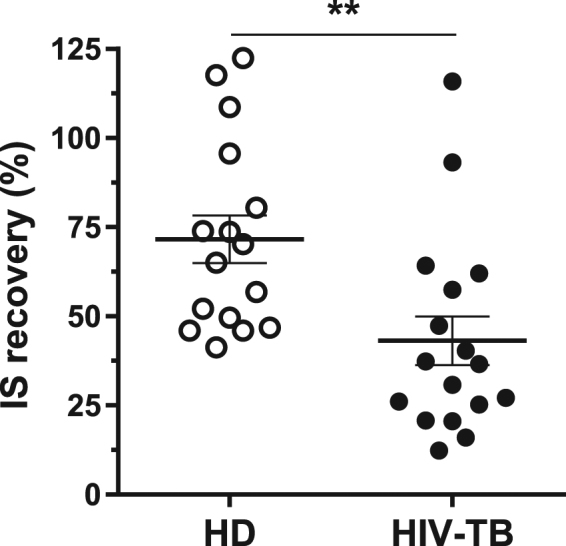
Percent of IS recovered from plasma samples. Results are shown for samples from HD (white circles, n = 16) and HIV-TB patients (black circles,n = 17). Each symbol represents an individual subject. Horizontal lines represent mean values and error bars symbolize the standard error of the mean (SEM). Wilcoxon matched-pairs signed rank test, **p < 0.01.

Ethyl acetate (EtOAc) is a widely used solvent in industry owing to its affordability and low toxicity^36^. Due to the polarity of oxygenated metabolites, we chose this solvent to perform liquid-liquid extraction, which allowed us to satisfactorily recover the compounds from a complex matrix. To assess extraction efficiency, we determined the relative and absolute recoveries of the evaluated steroids, as described below (Table 2). Recovery of the analytes was analyzed by comparing the results from pooled plasma and from fortified pooled plasma after the extraction procedure. Results displayed adequate relative recovery values, while absolute recovery of the analytes declined to a similar extent for all compounds (Table 2)^37^. These data indicate that the chosen IS had a similar behavior to the analytes and that the matrix effect in the extractive process was similar for all the compounds. Also, we estimated process efficiency (PE) as the ratio of the response of an analyte spiked before extraction and the response of the same analyte in MeOH, which represents the combined effects of extraction recovery and matrix. We found that PE ranged from 101% to 109%, which indicates satisfactory results in the overall procedure (Table 2)^37^. Surprisingly, during the processing of samples, we observed discrepancies in the absolute recovery between HD and HIV-TB when quantifying IS in the context of a calibration curve in MeOH (Fig. 2). These statistically significant differences may indicate variations in matrix complexities, which we corrected through the use of an IS during sample processing.

### Application of HPLC–MS/MS analysis to human plasma samples: biological relevance of the steroids studied

**Figure 3:**
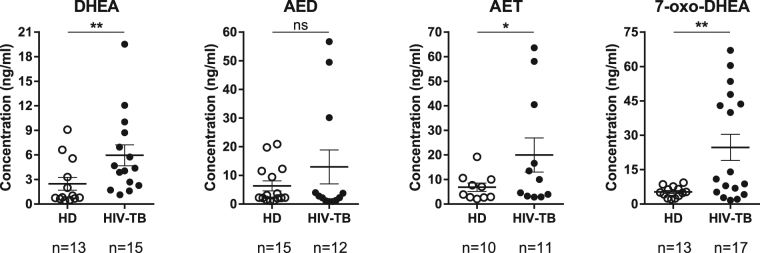
HPLC-MS/MS-based approach application to human plasma samples. Plasma concentrations of DHEA, AED, AET and 7-oxo-DHEA measured by HPLC-MS/MS for HD (white circles) and HIV-TB patients (black circles). Each symbol represents an individual subject (n values are shown). Horizontal lines represent mean values and error bars symbolize SEM. Mann-Whitney U test,*p < 0.05, **p < 0.01.

With the objective to study plasma levels of DHEA, AET, AED and 7-oxo-DHEA, HPLC-MS/MS was performed on samples from HD and a group of HIV-TB co-infected patients. Cohorts recruited in the current study did not present disparities in age or female/male distribution but there were significant differences in median CD4+ T cell counts, as expected (Table 3). We therefore report for the first time the simultaneous measurement of steroids DHEA, AET, AED and 7-oxo-DHEA in HIV-TB individuals by HPLC-MS/MS and their comparison with HD. Most interestingly, we observed statistically higher levels of DHEA, AET and 7-oxo-DHEA in HIV-TB, while no such result was found for AED (Fig. 3).

**Table 3:** Characteristics of the subjects enrolled. IQR: interquartile range. Mann-Whitney U test, **p < 0.01.

	HD (n = 16)	HIV-TB (n = 17)
Median age in years (IQR)	30.0 (28.0–36.5)	34.5 (32.2–41.0)
Female/male distribution	6/10	4/13
Median CD4+ count (IQR)	607.0 (490.5–767.0)	117.2 (72.2–357.1)**
Median Viral Load (IQR)	N/A	39,449 (845–81,475)

**Figure 4:**
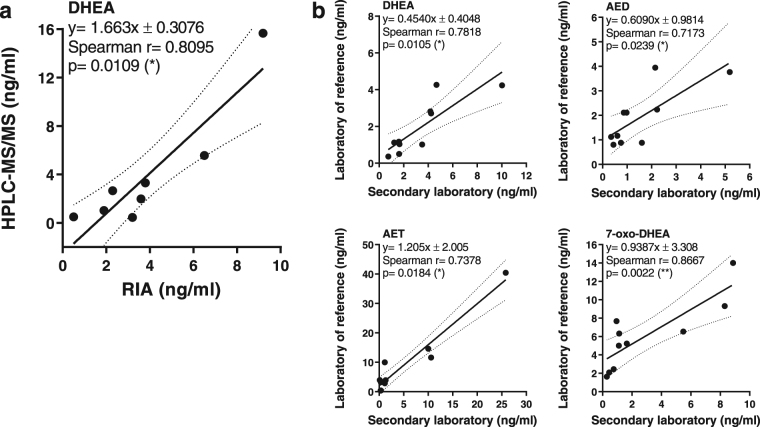
Comparison tests performed with a fully validated method and interlaboratory approach. **(a)** Correlation curve of DHEA plasma levels determined by HPLC-MS/MS and RIA from HD samples. Number of pairs XY = 8. (**b)** Interlaboratory reliability through a cross-validation assay. DHEA, AED, AET and 7-oxo-DHEA were determined both in the laboratory of reference and the secondary laboratory, and then the results obtained were compared by correlation analysis. Number of pairs XY = 10 (HD = 4, HIV-TB = 6). Solid lines represent linear regression curves and dotted lines symbolize 95% confidence intervals. Spearman’s rank correlation coefficient, p value and slope are indicated. *p < 0.05; **p < 0.01.

Afterwards, we aimed to compare the plasma steroid concentration determined by HPLC-MS/MS with that obtained by RIA and, alternatively, with the method described here performed in a different laboratory, employing different equipment and operator. First, we contrasted the results from HD samples with previous data obtained from an immunologically based method^12^ (Fig. 4a). Statistical analysis indicated that Spearman’s rank coefficient was over 0.8 (p < 0.005), which supports the idea that the degree of agreement between our approach and RIA could be clinically acceptable, even though more biological samples are needed to validate this result. Then, we tested interlaboratory reliability through a cross-validation assay. Plasma samples from disease-affected and non-affected individuals were included for comparison (Fig. 4b). We demonstrated a statistically significant correlation between the values from the laboratory of reference and the secondary laboratory, without differences among the data obtained (Mann-Whitney U test).

**Figure 5:**
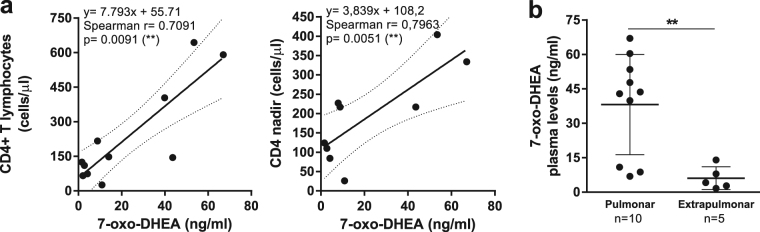
7-oxo-DHEA plasma levels are associated with a favorable clinical outcome. (**a**) 7-oxo-DHEA plasma levels positively correlated with absolute CD4+ T cell count (number of pairs XY = 11) and the CD4+ T cell nadir values (number of pairs XY = 9) in HIV-TB patients. Solid lines represent linear regression curves and dotted lines symbolize 95% confidence intervals. Spearman’s rank correlation coefficient, p value and slope are indicated. (**b**) HIV-TB patients with pulmonar TB exhibited higher 7-oxo-DHEA concentrations compared to those with extrapulmonar TB. Each symbol represents an individual subject (n values are shown). Horizontal lines represent mean values and error bars symbolize SEM. Mann-Whitney U test. ** p < 0.01.

Finally, we contrasted the levels measured for each hormone with clinical data from the patients enrolled: sex, age, absolute CD4+ T and CD8+ T cell counts, CD4:CD8 cell ratio, CD4+ T cell nadir value, viral load, and localization of TB. Interestingly, we observed significant associations for 7-oxo-DHEA, not being able to find the same results when analyzing other steroids. Figure 5 shows the relationship between plasma concentration of 7-oxo-DHEA and the values of absolute CD4+ T cell count and the lowest ever CD4+ T lymphocyte count (CD4+ T cell nadir). Moreover, higher concentrations of 7-oxo-DHEA were associated with the development of pulmonar TB, while lower levels were related to extrapulmonar TB.

## Discussion

In the current study, we propose a novel HPLC-MS/MS-based approach to simultaneously measure DHEA, AED, AET and 7-oxo-DHEA in human plasma. HPLC–MS/MS has been used in clinical laboratories during the last 10–15 years, and steroids were among the first molecules to be quantified using this technology^38^. Triple quadrupole mass spectrometry has emerged as a tool for improving detection sensitivity and resolution, which results in a simultaneous qualitative and quantitative measurement. These features have added versatility to the analysis of several analytes in a small volume of sample^38–40^. Nonetheless, developing a protocol to quantitatively assess an analyte requires availability of standards to quantify each molecule and systematizing a procedure for efficient extraction recovery.

The measurement of analytes without derivatization is increasingly feasible due to the development of highly sensitive mass spectrometry equipment, achieving lower limits of quantitation^41–43^. The LOQs achieved in this work are comparable to those informed by other authors under similar conditions^44^. Better results have only been possible when a different ionization source was used or analytes were derivatizated^44–46^. Ideally, reaction of derivatization should be total, fast, and reproducible, without chemical artifact generation. Derivatization usually improves sensitivity, facilitating the ionization procedure, but these results are difficult to accomplish in complex matrices for polyhydroxylated compounds, as AED and AET, which can yield two or more products.

Regarding the transitions reported for each analyte, we detected only one fragment ion to quantify AED. In our assays, a wide range of collision energy values were tested in order to find fragment ions and to select the most abundant. As described before, several compounds show extremely high molecule stability, making it difficult to generate more than one fragment ion in the collision cell^47,48^. Furthermore, we resolved plasma sample components using an HPLC column before analytes were introduced into the mass spectrometer. Through this procedure, it was also possible to identify AED by its retention time.

Interfering peaks which co-elute with the analytes of interest can cause ionization suppression, thus impairing detection sensitivity^49^. We estimated this matrix effect (ME) by comparing analyte MS/MS response in a MeOH solution and a pooled plasma extract, obtaining similar behaviors, with results allowing the use of external calibration for analyte measurement in plasma samples. Taking into account that we would need a large volume of plasma to build the calibration curve, this procedure would not be applicable in the clinic. In addition, as we showed in Fig. 2, the differences in the composition of the matrix of HD and coinfected patients would complicate the analysis. As a result, we decided to use MeOH to prepare the standard curve. Thus, the MeOH curve proved precise and accurate in quantifying the analytes in the matrix studied. Despite this, some samples were also analyzed using pooled plasma as matrix curve, also finding significant differences for DHEA, AET and 7-oxo-DHEA between HD and HIV-TB (data not shown).

To avoid matrix effects during sample preparation, we used an IS added at a concentration known prior to extraction^50–52^. If possible, the IS should be stable, with chromatographic and mass spectrometric ionization behavior comparable to that of the analytes. With the purpose of choosing an appropriate IS to be employed, preliminary experiments were carried out with the commonly used deuterium label, adding DHEA-16,16-d2 before extraction. Our results indicate that a significant loss of the deuterium label occurred (data not shown), as previously reported by other authors^53,54^. Deuterium loss from the standards due to biological reactions or chemical processes could compromise the accuracy of results, as it hinders reproducibility and may lead to erroneous concentration reports^53,55^. Even though the synthesis of stable 3α deuterium-labeled steroids has been reported^56^, obtaining these compounds is an extra step which increases the cost, time and complexity of the technique. Therefore, we decided to use a commercial IS with chemical and physical properties similar to those of the natural analytes and which is not present in the matrix studied, in order to relativize recovery to extraction efficiency.

When sensitivity is an issue, some reports propose the use of liquid-liquid extraction to enrich the sample with respect to the analyte and eliminate proteins and other hydrophilic metabolites^57,58^. A common problem in bioanalytical methods is that matrix effect may also alter recovery efficiency^37^. We calculated relative recovery and obtained satisfactory performances for all compounds, although we discovered variances between plasma samples from HIV-TB individuals and HD when absolute recovery was evaluated. This fact might be explained by differences in components of the matrix which interfere with the extraction process^50^. Chronic infections are known to involve an abnormal physiological concentration increase in protein and lipids. HIV-infected people develop multiple metabolic syndromes which are related to HIV-modulated and inflammation-related proteins, which enhance fatty acid synthesis, increase triglycerides levels, dysregulate lipid transport and alter cellular lipid metabolism^59,60^. Likewise, patients affected with TB present higher plasma levels of cytokines, C-reactive protein, matrix metalloproteinase-8 and alpha-1-antitrypsinin parallel with cortisol, estradiol, prolactin, growth hormone and thyroid hormone increases^61–63^. As in HIV-TB patients there is also an up-regulation of several proteins and lipid synthesis^64^, we expected consequent changes in matrix composition which might explain the differences found in absolute extraction efficiency^58^. Given matrix complexity in the context of co-infection, we believe the use of an IS may be convenient to correct matrix interferences and to obtain accurate results.

Summarizing, our approach involves a liquid-liquid extraction, the use of a commercial steroid added before extraction as IS and no derivatization techniques. Taking into account the above and according to the current guidelines^35^, we consider this methodology sensitive and precise for simultaneous quantifications of the metabolites in study. Future work will continue a thorough validation of this chromatographic method for the separation and quantitation of these and other significant steroids.

The assay developed allowed us to determine the concentrations of DHEA, AED, AET and 7-oxo-DHEA in human plasma samples. We used HD samples to evaluate the performance of the novel HPLC–MS/MS approach compared with RIA^12^, a validated radioimmunoassay. The results showed correspondence between both methodologies, obtaining a statistically significant Spearman’s rank correlation coefficient of 0.8095. Additionally, we correlated the results from HD and HIV-TB samples determined in a reference laboratory with those obtained in a secondary laboratory, and found that regression curve slopes slightly differed among metabolites. Considering the results from this analysis (see Fig. 4), we conclude that the values in the secondary laboratory were similar to those acquired in the laboratory of reference. In contrast, the curve slopes of DHEA and AED were <1.0 (0.4540 and 0.6090, respectively), indicating that the values from the secondary laboratory were lower.

It was previously observed that DHEA plasma levels measured by RIA are significantly diminished in HIV,TB and HIV-TB patients, compared to HD^10,12,65–68^. Moreover, antituberculous therapy seems to increase DHEA in plasma, restoring its concentration to the levels observed in healthy individuals^65^. Nevertheless, as shown before (Fig. 3), we observed that the HIV-TB cohort exhibited not only higher DHEA plasma levels, but also higher AET and 7-oxo-DHEA concentrations compared with HD. As discussed above, HIV-TB co-infection is a chronic inflammatory condition which dysregulates the production of cytokines and other compounds secreted by cells and tissues, as hormones or acute-phase proteins^11,69^. Therefore, we propose that the discrepancy between plasma DHEA concentration obtained by HPLC-MS/MS and RIA might be caused by differences in extraction efficiency due to matrix complexities, which can be solved with the use of an IS. As the radioimmunoassays used in clinical practice do not take advantage of including standards in their methodology, this generates a problem in sample preparation which cannot be solved.

Understanding the complex changes taking place during HIV-TB co-infection might lead to the identification of prognostic markers and the development of novel drug therapies. It is known that in HIV or TB, clinical data are predictors of the immune response against pathogens and a successful or unfavorable disease outcome^70,71^. Therefore, we contrasted plasma hormone concentrations with clinical data from patients, with remarkably interesting results obtained for 7-oxo-DHEA. On the one hand, we observed a positive and statistically significant correlation between the levels of this hormone levels and absolute CD4+ T cell count or CD4+ T cell nadir values in HIV-TB patients. On the other hand, we discovered that higher concentrations of 7-oxo-DHEA were associated with the development of a restricted pulmonar TB instead of disseminated infection.

First, the peripheral CD4+ T cell count is a standard measure of disease progression in HIV-infected individuals, since a higher CD4+ T cell count is associated with a lower short-term risk of AIDS events and higher body mass index^72,73^. Furthermore, HIV+ individuals with fewer peripheral CD4+ T cells are more prone to developing TB and exhibiting higher viral titers^69,74^. In the context of TB, it is well established that CD4+ T cells are critical for resistance to *Mtb*, and a functional deficiency of CD4+ T lymphocytes results in impaired granuloma formation, leading to failure restricting bacteria replication^75,76^. Additionally, higher nadir CD4+ T cell counts are related with favorable long-term outcomes and specific CD4+ and CD8+ T cell responses in HIV-1-infected individuals^70,72^. Second, clinical manifestations of TB are variable and dependent on a number of characteristics of the host immune system. Before the advent of the HIV pandemic, most of the TB cases reported were limited to the lungs, but an increase was later observed in the frequency of extrapulmonar TB in people whose immune function was compromised^77^. The frequency of TB (pulmonar as well as extrapulmonar) in subjects infected with HIV rises with the advance of immunosuppression^78^. In line with this, an epidemiological study has shown that people with pulmonar TB present higher CD4+ lymphocyte counts than those with extrapulmonar TB, indicating an immune system failure in pathogen control^78^.

To the best of our knowledge, this is the first report showing the simultaneous measurement of DHEA, AED, AET and 7-oxo-DHEA in plasma from HIV-TB individuals using HPLC-MS/MS technology. Our findings about 7-oxo-DHEA suggest that this DHEA metabolite with weak estrogenic and androgenic activities^31–33^ may be involved in the development of an effective immune response against *Mtb* and/or HIV in co-infected individuals. Generally, patients who fail to establish an adequate response against specific pathogens need the use of combined traditional and adjuvant therapies, and steroids are among the immunomodulatory compounds most commonly used^79–81^. Therefore, the results originated in this research work encourage us to develop more comprehensive studies regarding the role of these hormones in the pathology of HIV-TB co-infection.

## Methods

### Chemicals

DHEA (Fig. 1) (purity 99%), internal standard (IS) 16-methylene-17α-hydroxypregnenolone 3-acetate (Fig. 1) (purity 99%), sodium borohydride powder (NaBH_4_, purity ≥ 98.0%), lithium hydroxide, (LiOH, grade 98%), acetic anhydride (Ac_2_O, purity ≥ 99%), tertbutyl hydroperoxide (TBHP, solution 70% in H_2_O), Cerium (III) chloride heptahydrate (CeCl_3_, 99.9% trace metals basis), sodium chlorite (NaClO_2_, technical grade 80%), sodium sulfate (Na_2_SO_4_, ≥ 99.0%, anhydrous, granular), and formic acid (grade ≥ 95%) were purchased from Sigma-Aldrich Chemical Co (St. Louis, Missouri, USA). Solvents dichloromethane (CH_2_Cl_2_), methanol (MeOH), tetrahydrofuran (THF), pyridine (PY), ethyl acetate (EtOAc), hexane (HEX) and acetonitrile (ACN) were obtained from SINTORGAN S.A. (Buenos Aires, Argentina). All solvents and reagents were of analytical grade, with the exception of those used in HPLC-MS/MS, which were HPLC grade.

### Synthesis

All synthetic compounds were purified by column chromatography on silica and their NMR spectra were recorded on a Bruker AM-500 spectrometer (500 MHz for ^1^H and 125.1 MHz for ^13^C). The grade of purity achieved was > 99%.

AED (Fig. 1) was synthesized from DHEA by a reduction reaction with NaBH_4_ as previously described^82^, but solvent was modified by CH_2_Cl_2_:MeOH (1:4). The reaction was monitored by TLC and completed within 60 min. The solvent was evaporated *in vacuo* and the residue was taken in EtOAc and purified by column chromatography (HEX/EtOAc gradient).

7-oxo-DHEA (Fig. 1) was obtained from DHEA through three steps of synthesis. First, 1 equivalent of DHEA and 4 equivalents of acetic anhydride were dissolved in pyridine during 20 hours at room temperature to obtain 3-AcO-DHEA. Then, this compound was oxidized according to Silvestre and Salvador^83^ using NaClO_2_ and TBHP. Finally, the product was hydrolyzed with KOH in MeOH:H_2_O (8:2) during 2 hours at room temperature, extracted with EtOAc and purified by column chromatography (HEX/EtOAc 1:1).

AET (Fig. 1) was synthetized utilizing previously described conditions of Luche reduction^84^ from 3-AcO-7-oxo-DHEA, an intermediate compound in the synthesis of 7-oxo-DHEA. Then, the hydrolysis of 3-AcO group was achieved with LiOH. The crude mixture was purified by column chromatography (HEX/EtOAc1:1) to give the final product.

### Study subjects

In the current study, we recruited 33 subjects (Table 3) which were classified into two groups: 1) healthy donors (HD), with no history of TB, HIV or systemic infections; 2) HIV–TB patients, chronically HIV-1^+^ infected with active TB who received none or less than one week of anti-TB therapy. HIV diagnosis was determined by ELISA and confirmatory Western blot, while TB was detected through identification of acid-fast bacilli in sputum, a positive culture of TB bacilli and/or radiological data. Some HIV–TB individuals were on anti-retroviral treatment following the current guidelines^85^. None of the subjects had metabolic or endocrine disorders or received DHEA or glucocorticoids. Patients were evaluated at Hospital J.A. Fernández, Buenos Aires, Argentina. The investigation was approved by the Ethics Committee from Fundación Huésped, Buenos Aires, Argentina. Written informed consent was obtained from all participant subjects. In addition, all experiments were performed in accordance with current guidelines and regulations.

### Sample preparation

Plasma samples were obtained by centrifugation from peripheral blood collected during the morning, stored at −80 °C in plastic tubes and thawed at the time of extraction. One ml of plasma was centrifuged for 3 minutes at 4000 RPM in order to remove residual cells and particulate matter. Afterwards, plasma was transferred into a glass tube and the residue was washed with 250 μl of distilled water, it was centrifuged again and the supernatant was added to the plasma and spiked with IS (Fig. 1) at a final concentration of 40 ng/ml.

In order to extract unconjugated steroids, plasma samples were mixed with 2 ml of EtOAc, vortexed for 30 seconds and after 10 minutes the organic layer was transferred into another glass tube. This procedure was repeated 2 more times with 1.5 ml of EtOAc. The pooled organic layer was dried with 300 mg of sodium sulfate, the supernatant was filtrated and the solvent was evaporated *in vacuo*. The residue was taken in MeOH and filtrated with a syringe filter Millex-LG (0.20 µm, PTFE hydrophilic, 13 mm. Millipore, Darmstadt, Germany). The resulting filtrate was collected into an autosampler vial with inserts (MRQ™ Vials w/RSA™ Glass-12 × 32 mm, 1.2 ml) and, after removal of the solvent with a nitrogen stream, the extract was dissolved in 100 μl of MeOH before injection into the liquid chromatograph. Subsequently, the area acquired for each metabolite was relativized to the area of IS in order to calculate the concentration.

### Calibration standard and quality controls (QC)

Calibration standards and QC were prepared spiking with 10 μl of a stock solution of standards at MeOH in 1 ml of: (1) MeOH (solvent) or (2) a pool of plasma from HD (pooled plasma). Curves included seven calibration levels at a final concentration of 3, 10, 30, 100, 300, 1000 and 3000 ng/ml. We used three replicates for each level of the calibration curves. Additionally, each point was spiked at 40 ng/ml of IS and processed as described above. The curves were obtained by plotting the “peak area of standard compound/peak area of IS” ratio (*response*) against the analyte spiked concentration. QC were prepared in the same way as calibration standards at 3 ng/ml as the lower limit of quantitation (LLOQ), 10 ng/ml as lower QC (LQC) and 3000 ng/ml as higher QC (HQC).

Limit of detection (LOD), limit of quantitation (LOQ), linearity, accuracy, precision (intra and interday), recovery and process efficiency were calculated (Table 2). In order to calculate the LOD and LOQ, a solution of standards in MeOH was used. Serial dilutions (from 300 to 3 ng/ml) of the standards in MeOH were prepared and injected into the mass spectrometer in decreasing order. From the response of each analyte concentration, the “signal-to-noise” ratio (S/N) was calculated. LOD was defined as a S/N superior to 3, while LOQ was determined as the lowest concentration at which S/N ratio was greater than 10^34,86–88^.

Precision and accuracy were calculated for LLOQ, LQC and HQC. Accuracy was calculated as the mean relative error (RE%) of 5 independent determinations in different days, while precision was assessed as the coefficient of variation (CV%) of 5 independent determinations during the same day (intraday) or 5 measures on different days (interday).

With the intention of studying matrix effect (ME), the recovery (R) and the efficiency of the process (PE), three different solutions fortified with standards at 30 ng/ml and IS at 40 ng/ml were prepared: (**A**) MeOH solution; (**B**) Extracted pooled plasma, spiked prior to HPLC injection; (**C**) Supplemented pooled plasma, subsequently submitted to the extraction procedure. Matrix effect was evaluated in two ways: (1) Contrasting the curve slopes of solvent and fortified pooled plasma calibration curves^34,89^; (2) Comparing the *response* in triplicate from **A** with **B** calculated as: ME%: **B**/**A** × 100.The relative and absolute extraction recoveries were evaluated comparing in triplicate **B** and **C**. The calculation of relative recoveries was based on the formula: Relative R (%) = *response* from **C**/*response* from **B** × 100. Absolute recovery was calculated as: Absolute R (%) = analyte area from **C**/analyte area from **B** × 100. Process efficiency was estimated by multiplying the ME by the R, expressing the ratio of the *response* of an analyte spiked before extraction (**C**) and the response of the same analyte in a MeOH solution (**A**)^37^.

### HPLC-MS/MS conditions

An interlaboratory comparison with subject samples was conducted at two laboratories: (1) laboratory of reference, where this approach was developed; (2) secondary laboratory, where the results of analyte quantitation were compared by a correlation analysis.

The equipment owned by the laboratory of reference was acquired from Thermo Fisher Scientific Inc. (Waltham, Massachusetts, USA). Separation and quantification of steroids was performed using an Ultimate 3000 RSLC Dionex HPLC coupled with a TSQ Quantum Access Max triple quadrupole mass spectrometer. Electrospray ionization (ESI) in positive ion mode with a heated electrospray ionization (HESI-II) probe and method of multiple reaction monitoring (MRM) were used for performing mass spectrometric quantitation. The conditions were as follows: 25 psi sheath gas (nitrogen), 5 psi auxiliary gas (nitrogen), 1.5 mTorr collision gas (argon), 5000 V ion spray voltage and 200 °C vaporizer temperature.

Chromatographic separation was carried out on a Hypersil GOLDC18 1.9 μm (50 × 2.1 mm) column at 25 °C with an injection volume of 6 μl. Mobile phase consisted of water (solvent A) and ACN (solvent B), both with 0.1% formic acid as additive to improve electrospray ionization^90^. The flow rate was set at 0.20 ml/min. Running started with a linear gradient of solvent B from 20% to 30% in 10 minutes, then a plateau until minute 20, a linear gradient to 95% for 2 minutes, a 4 minute plateau and finally a gradient to 20% until minute 28, ending with a re-equilibration step for 4 minutes. This method allowed separation of all compounds within 32 min. LC separation combined with tandem MS spectrometry enabled the differentiation of each compound by their retention times and MRM transitions (Table 1). Data acquisition was analyzed with Thermo Scientific™ Xcalibur™ software.

The cross-validation assay was performed in a secondary laboratory with equipment purchased from Agilent Technologies (Santa Clara, CA, USA). UHPLC analysis was performed on a 1290 Infinity II LC with a ZORBAX Rapid Resolution High Definition SB-C18 2.1 × 50 mm (1.8 μm) column at 25 °C and an injection volume of 5 μl. Flow rate was set at 0.220 ml/min and the elution gradient was optimized for this equipment, using the same mobile phase. Running began with a linear gradient from 20% to 40% solvent B in 6 minutes, then a plateau of 4 minutes, a linear gradient to 95% solvent B until minute 12 followed by a 5-minute plateau, another gradient to 20% B for 2 minutes and a re-equilibration step to the initial conditions for 2 minutes. This optimized method allowed the separation of compounds within 20 min. Quantification was carried out in an Agilent 6460 Triple Quadrupole LC/MS with Standard ESI Source under the same conditions specified above (Table 1) and analyzed with MassHunter Workstation software.

### Statistics

Statistical analyses were conducted using GraphPad Prism 6 software. Matrix effects were evaluated by comparing solvent and matrix calibration curves by the standard-addition method^34,89^. The comparison of measurements by LC-MS/MS with radioimmunoassay (RIA, Packard Cobra II Gamma Counter, Packard, Meriden, CT, USA)^12^, as well as the interlaboratory study, were analyzed through Spearman’s rank correlation coefficient. Comparisons between groups (HD and HIV-TB) were performed using the non-parametric Mann–Whitney U test. For all evaluations, a p value < 0.05 was considered significant.

## Electronic supplementary material

Supplementary Figure S1
