# Supplementary material for: Determination of dehydroepiandrosterone and its biologically active oxygenated metabolites in human plasma evinces a hormonal imbalance during HIV-TB coinfection
Source: Sci Rep. 2018 Apr 27;8:6692. doi: 10.1038/s41598-018-24771-8 (PMC5923237; doi:10.1038/s41598-018-24771-8)
Supplement: Supplementary file 1 — Supplementary Figure S1 [file 41598_2018_24771_MOESM1_ESM.docx]

**Determination of dehydroepiandrosterone and its biologically active oxygenated metabolites in human plasma evinces a hormonal imbalance during HIV-TB coinfection.**

*María Belén Vecchione, Javier Eiras, Guadalupe Verónica Suarez, Matías Tomás Angerami, Cecilia Marquez, Omar Sued, Graciela Ben, Héctor Miguel Pérez, Diego Gonzalez, Patricia Maidana, Viviana Mesch, María Florencia Quiroga and Andrea Claudia Bruttomesso.*

*
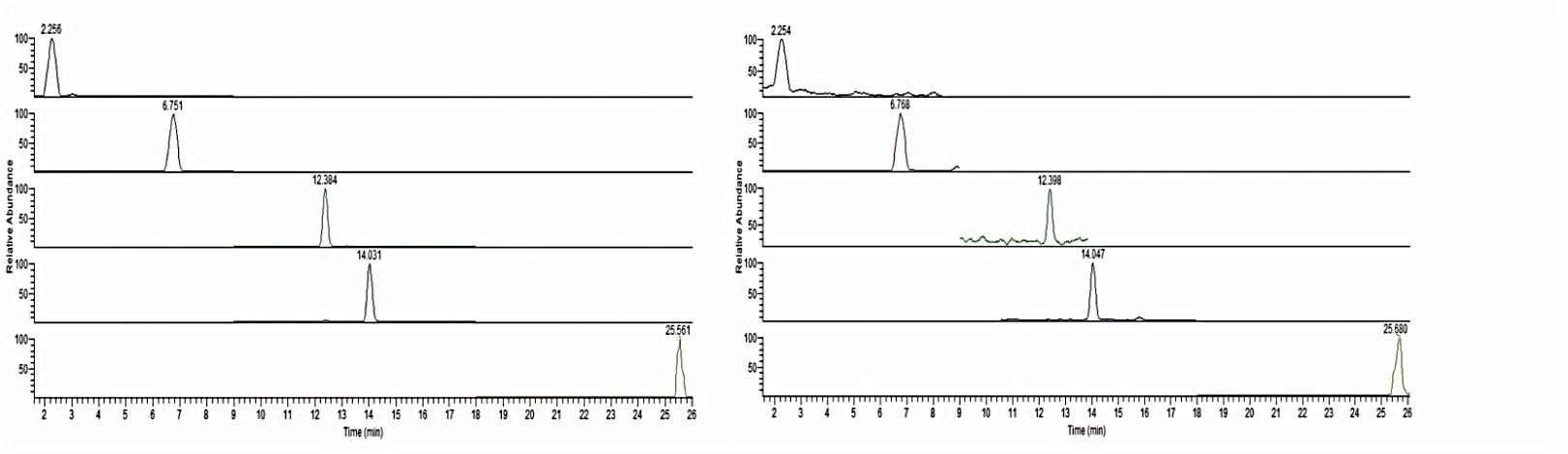
*

**Figure S1.** Representative chromatograms of a solution of standards at 30 ng/ml in MeOH (a) and endogenous levels of steroids in plasma (b), both spiked with 40 ng/ml of IS. Relative abundance (Y axis) and time (X axis) are represented. Order of elution: AET, 7-oxo-DHEA, AED, DHEA and IS.
